# *Pediococcus pentosaceus*: Screening and Application as Probiotics in Food Processing

**DOI:** 10.3389/fmicb.2021.762467

**Published:** 2021-12-16

**Authors:** Yining Qi, Le Huang, Yan Zeng, Wen Li, Diao Zhou, Jianhua Xie, Junyan Xie, Qiang Tu, Dun Deng, Jia Yin

**Affiliations:** ^1^Key Laboratory of Protein Chemistry and Developmental Biology of Fish of Ministry of Education, Hunan Provincial Key Laboratory of Animal Intestinal Function and Regulation, Hunan International Joint Laboratory of Animal Intestinal Ecology and Health, Hunan Normal University, Changsha, China; ^2^Tangrenshen Group Co., Ltd., Zhuzhou, China; ^3^CAS Key Laboratory of Agro-ecological Processes in Subtropical Region, Hunan Provincial Key Laboratory of Animal Nutritional Physiology and Metabolic Process, National Engineering Laboratory for Pollution Control and Waste Utilization in Livestock and Poultry Production, Institute of Subtropical Agriculture, Chinese Academy of Sciences, Changsha, China; ^4^CAS Key Laboratory of Quantitative Engineering Biology, Shenzhen Institute of Synthetic Biology, Shenzhen Institutes of Advanced Technology, Chinese Academy of Sciences, Shenzhen, China

**Keywords:** *Pediococcus pentosaceus*, probiotics, food additives, antimicrobial activity, cell surface characteristics, resistance

## Abstract

Lactic acid bacteria (LAB) are vital probiotics in the food processing industry, which are widely spread in food additives and products, such as meat, milk, and vegetables. *Pediococcus pentosaceus* (*P. pentosaceus*), as a kind of LAB, has numerous probiotic effects, mainly including antioxidant, cholesterol-lowering, and immune effects. Recently, the applications in the probiotic- fermentation products have attracted progressively more attentions. However, it is necessary to screen *P. pentosaceus* with abundant functions from diverse sources due to the limitation about the source and species of *P. pentosaceus*. This review summarized the screening methods of *P. pentosaceus* and the exploration methods of probiotic functions in combination with the case study. The screening methods included primary screening and rescreening including gastric acidity resistance, bile resistance, adhesion, antibacterial effects, etc. The application and development prospects of *P. pentosaceus* were described in detail, and the shortcomings in the practical application of *P. pentosaceus* were evaluated to make better application of *P. pentosaceus* in the future.

## Introduction

Owing to the development of antibiotic resistance and the potential damage to human health by chemical food additives, there is a cumulative need for natural food additives (O’Connor et al., 2020). Lactic acid bacteria (LAB) have been used in a wide range of fields for thousands of years as the natural fermentors and producers of probiotic factors, such as food, medicine, and feed. Admittedly, they are a type of food-grade microorganisms non-toxicity and harmless for human (Ouwehand, 1999). Additionally, LABs, with efficient antibacterial, antioxidant, cholesterol-lowering, and immune activity, are available for improving food flavor and enhancing food nutrition in food processing (Erten et al., 2014). Given above advantages, LABs are progressively widespread in the processing of various food, including dairy products, bread, pickles, fruit and vegetable juice, etc. For example, the use of LAB bacteriocins is becoming prominent in meat preservation as the preservatives succedaneum including nitrite (Nanasombat et al., 2017). LABs are a good choice to maintain food flavor and avoid the loss of nutritional components in the products processing (Gutiérrez-Cortés et al., 2018). The genera of *Lactobacillus*, *Lactococcus*, *Leuconostoc*, *Pediococcus*, and *Streptococcus* are imperative members of LAB. The genus of *Lactobacillus* was commonly used as probiotics, since they were believed ideal members of the gut microbiota with a good safety profile in the previous study (Shokryazdan et al., 2017). However, the main focus was frequently on antibacterial activity in most existing studies and LAB were limited in food types and application as a natural food additive. Therefore, there is an accumulative need to find novel probiotics with well-probiotic activities.

Numerous studies confirmed that LAB had potential application value, including *Pediococcus pentosaceus* (*P. pentosaceus*). *P. pentosaceus* belongs to family *Streptococcaceae*, genus *Pediococcus*, and is a kind of LAB because it can produce lactic acid (Chen et al., 2020). The individual of *P. pentosaceus* is spherical in pairs or quadruplets, and an immotile bacterium belonging to facultative anaerobic Gram-positive bacteria. Previous study confirmed that *P. pentosaceus* was inherent in naturally fermented products and could have active functions in product quality, food safety, and production efficiency by preparing into a mixed fermentor with other bacteria (Balakrishnan and Agrawal, 2014; Jang et al., 2015; Gong and Qi, 2020; Montemurro et al., 2020; Xu et al., 2021). In addition, many studies reported that *P. pentosaceus* had probiotic functions including anti-inflammation, anti-cancer, antioxidant, detoxification, and cholesterol-lowering (Zhao et al., 2012; Thirabunyanon and Hongwittayakorn, 2013; Sellamani et al., 2016; Asami et al., 2017; Kim et al., 2019). However, the use of LAB poorly supports the diverse demands in food processing. More kinds of probiotic strains are in demand, and *P. pentosaceus* is the one that may make sense. Given the good application value and probiotic effect of *P. pentosaceus*, the identification and applications of *P. pentosaceus* in the food industry have attracted more attention.

To make better use of *P. pentosaceus* in the food industry and remain probiotic effects in human body to a large extent, several researchers from diverse countries had investigated on the probiotic potentials of *P. pentosaceus* in local-derived foods. It is known that the diversity of strains may contribute to dissimilar fermentation products with various probiotic functions. For instance, some subtypes of *P. pentosaceus* can produce common antibacterial substances, such as organic acids and inorganic substances with bacteriostasis effect, and a small number can conduce to the synthesis of substances which could inhibit the growth of other microorganisms, such as proteins, alcohols, and lipids (Shukla and Goyal, 2014; Sellamani et al., 2016; Nanasombat et al., 2017; Ghosh et al., 2019). According to the genomic information from 41 *P. pentosaceus* strains documented in NCBI, there was no obvious clustering, suggesting that high genetic diversity of the strains and the characteristic of specificity (Jiang et al., 2020). The screening methods for *P. pentosaceus* must be different and tailored based on individual characteristics and dissimilar product applications (Jiang et al., 2020). However, the limitation about the source and species of *P. pentosaceus* prompts us to screen strains well-probiotic activities from different sources.

**Figure 1:**
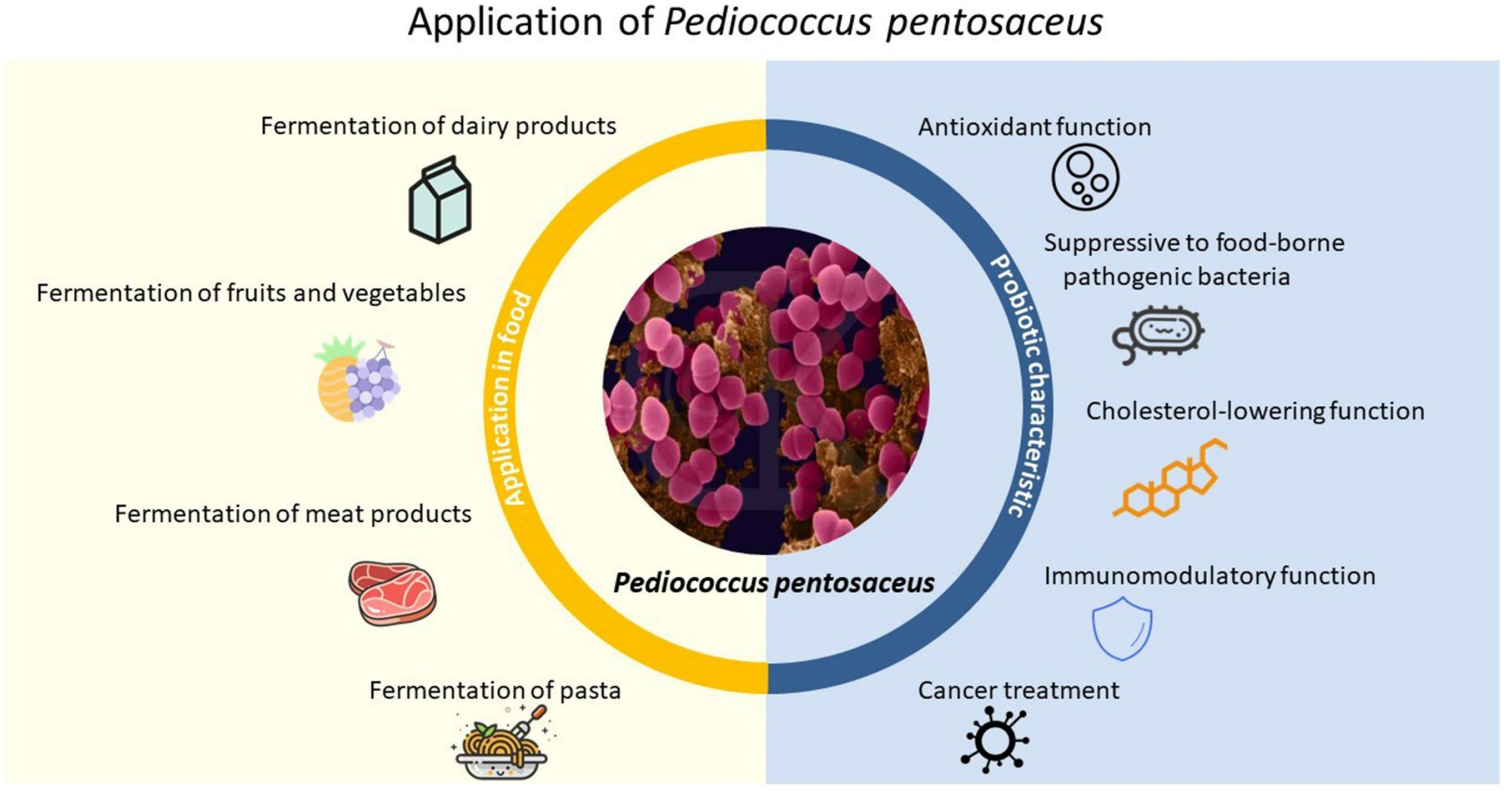
Application of *P. pentosaceus* in food processing and probiotic characteristic.

Although more and more studies have found that *P. pentosaceus* has very important uses, there is still a lack of systematic and pertinent articles on the screening and application of *P. pentosaceus*. Based on these situations, we have consulted a large number of literature and in order to better apply *P. pentosaceus* in various aspects and provide a comprehensive and concise description for researchers to conduct further research. In this review, the relevant researches were described in recent years, along with the analysis and summarization of the screening and e probiotic effects of *P. pentosaceus* involving a series of *in vivo* and *in vitro* studies. The obtained excellent strains could provide target strains for production practice and also bring more options for the preparation of new food additives in the future. Applications of *P. pentosaceus* in the food processing industry and its potential probiotic characteristic were also introduced (Figure 1).

## *P. pentosaceus* Strain Screening

### The Sources of *P. pentosaceus*

At present, researchers from different countries have isolated *P. pentosaceus* from different sources. The research area reviewed in this article includes 15 countries, and 65 strains of *P. pentosaceus* have been isolated and identified, of which the main source of isolation is food (53 strains, 81.6%), especially fermented food (41 strains, 63.1%). And most of the isolated *P. pentosaceus* were confirmed to have probiotic effects (Table 1). As a preferred source for separation, food-derived *P. pentosaceus* has the advantage of being safer and more stable. For example, *P. pentosaceus* in common fermented foods in daily life, such as pickles, dairy products, and sausages, are often used as fermentation initiators. However, the *P. pentosaceus* derived from human intestines or feces have less been studied, but they are likely to be more adapted to the human intestinal environment and exert better probiotic effects. This suggests that the sources of *P. pentosaceus* should be more extensive in the future.

**Table 1:** Different sources of *P. pentosaceus*.

	**Country**	**Subspecies of *P. pentosaceus***	**Probiotic characteristics**	**References**
Tsuda-Kabu pickle	Japan	*P. pentosaceus* QU 19	Potential hypoglycemic activity	Fujiwara et al., 2020
Onion	Korea	*P. pentosaceus* ON89A	Anti-inflammatory effect	Kwon et al., 2018
Fermented food Khadi	India	*P. pentosaceus* GS4 (MTCC 12683)	Purification and characterization of pediocin\cell adherence property	Ghosh et al., 2019
Pickled radish	China	*P. pentosaceus* strain L1	*In vitro* probiotic characteristics and inflammatory responses	Yin et al., 2020
Natural fermented cherry Juice and pickled pig’s ear	China	*P. pentosaceus* CCTCC AB2019253	Improve the characteristic organoleptic properties	Xu et al., 2021
Harbin dry sausages	China	*P. pentosaceus* R1	Enzyme-producing strain	Sun et al., 2019
Soil samples	China	*P. pentosaceus* SL001	A dietary probiotic in freshwater fish aquaculture	Gong et al., 2019
Cut grass	German	*P. pentosaceus* DSM 32291	A silage additive for all animal species	Rychen et al., 2018
Artisanal cheese	Brazil	*P. pentosaceus* ST65ACC	Aggregate with *L. monocytogenes*	Cavicchioli et al., 2019
Artisanal Minas cheese	Italy	*P. pentosaceus* 147	Improve bacteriocin activity	Gutiérrez-Cortés et al., 2018
Ready-to-eat pork ham	Brazil	*P. pentosaceus* ATCC 43200	Bacteriocin-like inhibitory substances (BLIS)	de Azevedo et al., 2020a
Corn silage	German	*P. pentosaceus* LBM 18	BLIS	de Azevedo et al., 2020b
Octopus jeotgal and radish kimchi	Korea	*P. pentosaceus* SC28	Bioactive properties for health	Yang et al., 2020
Traditional sour meat	China	*P. pentosaceus* SWU73571	Improve the quality and safety of sour meat	Zhang et al., 2020b
Fermentative vegetable pickles	Japan	*P. pentosaceus* AK-23	Neutralization of LPS	Asami et al., 2017
Yak-Kong (a small black soybean)	Korea	*P. pentosaceus* AOA2017	Antioxidant ability	Kim et al., 2019
Traditional vegetable pickles	Japan	*P. pentosaceus* NB-17	Stimulate immune activities and showed allergic inhibitory effects	Jonganurakkun et al., 2008
Raw milk artisanal cheeses	Brazil	*P. pentosaceus* ST65ACC	Isolate and characterize bacteriocin	Cavicchioli et al., 2017
Fermented vegetable beverage	Japan	*P. pentosaceus* strain IDS885	Treat mild to moderate ulcerative colitis	Bamba et al., 2018
Kimchi	Korea	*P. pentosaceus* T1	Antilisterial agent in fish products and a starter to control overmaturation of kimchi	Jang et al., 2015
Food isolates	Portugal	*P. pentosaceus* SB83	A vaginal probiotic	Borges et al., 2013
The faba bean flour	Italy	*P. pentosaceus* VTT E-153483	Contribute to faba bean dough processing	Rizzello et al., 2019
Rumen liquor of goat	India	*P. pentosaceus* MTCC 12613	Antibacterial activity toward *Listeria monocytogenes in vitro* bacteriocin	Ladha and Jeevaratnam, 2020
Rainbow trout intestine and trout fish feed	Argentina	*P. pentosaceus* RC001 and RC008	Use in rainbow trout culture	Martinez et al., 2017
Northeast pickled cabbage	China	*P. pentosaceus* PP04	Improved lipid metabolic disorder and oxidative stress response effectively	Wang et al., 2020
Kimchi	Korea	*P. pentosaceus* KFT18	Immunostimulating effects	Shin et al., 2016
Fermented cucumber	India	*P. pentosaceus* CRAG3	Probiotic property and glucansucrase-producing ability	Shukla and Goyal, 2014
Sunki pickle	Japan	*P. pentosaceus* Sn26	Anti-allergic effect	Masuda et al., 2010
Healthy volunteers.	China	*P. pentosaceus* LI05	Prevents CCl4-induced liver cirrhosis	Shi et al., 2017
Kombucha	Romania	*P. pentosaceus* L3	Candidates for the food industry	Diguță et al., 2020
Fermented seafood	Korea	*P. pentosaceus* FB145 and FB181	Biosorption of cadmium	Le and Yang, 2019
Raw and fermented pork products	Thailand	*P. pentosaceus* P0805	Meat starter cultures	Nanasombat et al., 2017
Beans	India	*P. pentosaceus* CFR B19	A natural preservative in various food products	Venkateshwari et al., 2010
Plant-derived	Japan	*P. pentosaceus* LP28	Antiobesity effect	Higashikawa et al., 2016
Fermented brown rice	India	*P. pentosaceus* CFR R123	Evaluate the fate of phytate and calcium solubility during fermentation	Raghavendra et al., 2011
Foal’s feces	Brazil	*P. pentosaceus* PP40,	Alternatives prophylactic treatments for *Salmonella Typhimurium* infection	Silva et al., 2017
Ryegrass	Italian	*P. pentosaceus* KCC-23	Anti-fungal, probiotic and antioxidant properties	Ilavenil et al., 2016
Fruits and fermented foods	Malaysia	*P. pentosaceus* Te010	Biopreservative in bakery products and other processed foods	Muhialdin et al., 2011
Cheeses	Denmark	*P. pentosaceus* KUH5 and KUH7	Histamine forming behavior	Møller et al., 2020
Traditional Foods	Malaysia	*P. pentosaceus* UL-2and UL-6	Extracellular proteolytic activities and capability of producing AAs	Toe et al., 2019
Vacuum-packaged meat	China	*P. pentosaceus* ACCEL	Bacteriocin	Wu et al., 2004
Fermented finger millet	Korea	*P. pentosaceus* KID7	Develop cholesterol-lowering functional food	Damodharan et al., 2015
Kimchi	Korea	*P. pentosaceus* K23-2	Natural biopreservatives pediocin	Shin et al., 2008
Split barley kernels	Finland	*P. pentosaceus* VTT E-90390	Enhancement of malt processability	Laitila et al., 2006
Traditional fermented dairy products	India	*P. pentosaceus* KX214298	The anti-fungal and ZEA inhibitory activity of PPCS against *F. graminearum*	Sellamani et al., 2016
Fermented fish and chicken	Thailand	*P. pentosaceus* strain PP 04	Control biofilms of food-borne pathogens	Tatsaporn and Kornkanok, 2020
Soymilk yogurt	Japan	*P. pentosaceus* MYU 759	Antioxidant capacity	Yamamoto et al., 2019
Fermented pork sausage	Thailand	*P. pentosaceus* HN8	Producing γ-aminobutyric acid (GABA)	Ratanaburee et al., 2013
Longan fruit	Japan	*P. pentosaceus* LP28	Reduce The Obesity and Fatty Liver	Zhao et al., 2012
Fermented sausages	German	*P. pentosaceus* LMQS 331.3	Contribute to product and consumer safety bacteriocin	Bungenstock et al., 2020
Pao cai	China	*P. pentosaceus* L1	Producing functional foods	Zhenhui et al., 2015
Traditional sourdoughs	China	*P. pentosaceus* strain DSM 20336	Original adjunct cultures in the steamed bread making process	Xing et al., 2019
Soil sample	India	*P. pentosaceus* EU569832	Biomedical applications	Ramos et al., 2020
Idly batter	India	*P. pentosaceus* VJ13	Beneficial probiotic in functional foods	Patel et al., 2010
Kimchi	Korea	*P. pentosaceus* T1	Natural anti-listerial agents	Jang et al., 2014

### Isolation and Culture and Preliminary Screening

The conventional screening method for probiotics is mainly based on different kinds of selective media, with a certain screening factor selected according to the use purpose. For example, *P. pentosaceus* with pathogen-suppressive effect can be screened by the inhibition zone method using an Oxford Cup, and strains with antioxidant effect can be screened by monitoring the survival outcome after a culture with an addition of H_2_O_2_ (Zhang et al., 2020a).

Currently, a combination of conventional screening and molecular biological identification is frequently used to comprehensively determine the type of probiotics from the morphological, biochemical and molecular levels. (Chen et al., 2020) obtained *P. pentosaceus* with *in vitro* antibacterial effect from sow milk. They firstly inoculated sheep milk samples into a culture medium, followed by purification of the single-strain isolates using the repeated streaking method; and agar diffusion assay was then conducted to test antibacterial activity; following that, DNA isolation and PCR amplification were performed using the universal primers, and the strains were identified by 16S rDNA gene sequence homology analysis.

For strains that exert probiotic effects by metabolites, additional growth curve and acid production curve analysis should be performed. The typical growth curve of microorganisms is divided into four periods: lag phase (adaptation phase), logarithmic phase, stable phase, and decay phase(Prescott). In the development of general microbial preparations, bacterial culture in the middle and late logarithmic phases and stable phases are often collected. Secondary metabolites, such as bacteriocins and extracellular polysaccharides, are often produced in the stationary phase. By understanding the growth curve of the strain, it is possible to cultivate a bacterial solution with an appropriate growth time. Yanfei et al. (2019) found that the growth rate of *P. pentosaceus* began to enter the logarithmic growth phase at about 4 h and reached a stable phase after 12 h of culture, and its growth slowed down. Moreover, during the adaptation and logarithmic phases of *P. pentosaceus*, the pH value in the culture medium has been declining, from 5.64 to 4.14, and stabilized at 12 h. It still maintains stable growth under acidic conditions, indicating that *P. pentosaceus* has strong acid-tolerant ability and can survive under lower pH conditions.

### Secondary Screening

**Figure 2:**
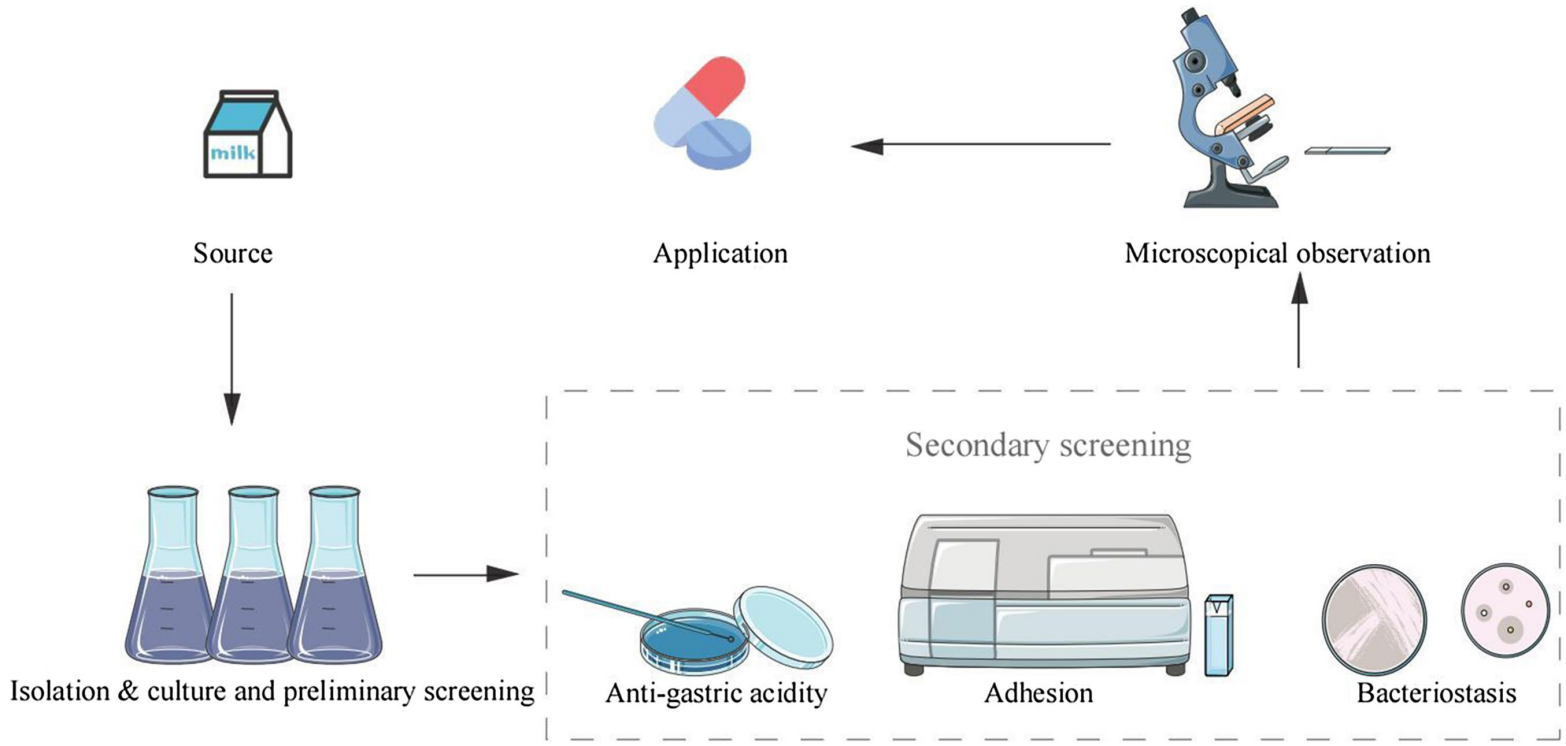
*P. pentosaceus* strain screening diagram.

There are some other requirements for probiotics being applicable. A list provided by Food and Agriculture Organization/World Health Organization (FAO/WHO) describes some commonly used *in vitro* tests for screening and identification of potentially applicable probiotic strains, including analyses in I) anti-gastric acidity; II) activity of bile salt hydrolase (BSH) and resistance to bile salt; III) adhesive ability to mucus and/or human epithelial cells and cell lines to decrease adherence of pathogenic bacteria; IV) antibacterial and antagonistic activity against potential pathogenic bacteria (Shokryazdan et al., 2017). It should be noted that these tests for screening are not specific to *P. pentosaceus*, but are for probiotics. However, in order to ensure safety and reliability, *P. pentosaceus* utilized in foods must meet the above requirements, a secondary screening is therefore required to exclude ineligible strains (Figure 2).

### Anti-gastric Acidity

A good ability to anti-gastric acid after ingestion is a prerequisite for probiotics to survive and colonize in intestinal tract (Martinez et al., 2017). In normal human body, the gastric acidity ranges from pH 1.5–4.5, which is sufficient to kill most of the ingested microorganisms (Xu et al., 2011). Studies reported that most LAB survived only 1 h in a pH 1.5 environment, while a majority remained to have a high survival ability within 3 h in a pH 2.0–3.0 environment (Jonganurakkun et al., 2008; Martinez et al., 2017; Fujiwara et al., 2020; Yin et al., 2020). Thus, a setting of pH 2.0–3.0 to simulate gastric juice is recommended in anti-acidity test to screen *P. pentosaceus* strains with acid tolerance.

### BSH Activity and Resistance to Bile Salt

In addition to gastric digestion, the digestion and transport by the small intestine is also an indispensable process for foreign bacteria to colonize and exert a role. Liver cell-derived bile salts destabilize cells with their effects on lipid dissolution and emulsifying in the digestive tract (Caggia et al., 2015). In normal people, the concentration of bile salts in the duodenum can reach 0.3–3 g/kg (Fitzgerald et al., 2000). A good tolerance to low pH of gastric juice and to bile salts in intestinal fluid for a certain period of time is one of the important criteria for selecting probiotics (Damodharan et al., 2015). Besides, despite the activity status in the intestinal tract, the tolerance to bile salts also affects the cholesterol-lowering function of probiotics (Argyri et al., 2013). The tolerance to bile salts varies with the type of probiotics, while the bile salt concentration remains to range between 0.1–1.0% (Ilavenil et al., 2016; Silva et al., 2017; Fujiwara et al., 2020; Tatsaporn and Kornkanok, 2020; Yang et al., 2020).

### Adhesion

Probiotic strains can adhere to gastrointestinal mucosa to survive and stably colonize and subsequently exert metabolic activities (such as production of digestive enzymes and organic acids). They can compete with the resident flora by producing antibacterial substances, and can also antagonize pathogenic bacteria, which plays a regulatory role in immune response (Yin et al., 2020). Given these characteristics, the adhesion of probiotics in the intestinal tract is generally used to indicate the applicable potential of probiotics.

In adhesion assay, the control strain usually selects the *Lactobacillus rhamnosus* GG (LGG), which is a commercial probiotic internationally recognized with strong adhesion ability. The colon cancer cell lines Caco-2 and HT-29 are widely used cell models *in vitro* at present (Kim et al., 2019; Bungenstock et al., 2020). Adhesion assays include: (1) Co-culture of bacteria and cells: i) co-culture of bacteria and cells; ii) washing, and removal of floating bacteria; iii) processing of the cells to blow out the adherent bacteria; and iv) calculating the number of live bacteria to compute adhesion rate. (2) Co-culture of bacteria and cells plus staining process: i) co-culture of bacteria and cells; ii) washing, and removal of floating bacteria; iii) fixation with methanol; iv) Gram’s or Giemsa’s staining; v) microscopic observation and adhesion rate computation (Raghavendra et al., 2010; Shukla and Goyal, 2014; Damodharan et al., 2015; Dubey et al., 2020).

It is reported that the strains with high self-aggregation ability also have high adhesion. In addition, the self-aggregation property could facilitate probiotics to form biofilm and colonize in the host intestinal tract, which could prevent the adhesion and invasion of pathogenic bacteria, showing a possible correlation with pathogen clearance (Rujing et al., 2019). Likewise, higher hydrophobicity also correlates to a higher adhesion ability in the intestinal tract (Jin et al., 2020). Probiotic strains are required to have at least 40% hydrophobicity (Re et al., 2010).

### Bacteriostasis

Antibacterial activity is an important indicator that reflects the ability of probiotics to competitively kill or suppress harmful microorganisms in the intestinal tract (de Azevedo et al., 2020a). *Pseudomonas aeruginosa*, *Escherichia coli*, *Staphylococcus aureus*, and *Salmonella*, etc. are used as pathogen indicators in testing the antibacterial activity of probiotics (Ghosh et al., 2019; Ladha and Jeevaratnam, 2020).. Assays include: (1) Oxford Cup method: (i) plate coated by different indicator bacteria and transferred to an Oxford Cup; (ii) addition of the bacterial supernatant and culture; (iii) determination of antibacterial activity according to the diameter of the inhibition zone. (2) Two-layer plating method: (i) addition of bacterial culture medium in a plate; (ii) chlorine gas processing; (iii) spot inoculation of the soft agar (containing indicator bacteria) to the surface of the modified plate; (iv) measurement of the inhibition zone diameter. (3) Agar diffusion method: (i) addition of the soft agar (containing indicator bacteria) to an agar plate, hole punch; (ii) inoculation of the bacterial supernatant or suspension into the holes; (iii) measurement of the inhibition zone diameter after 48 h of culture (Zhenhui et al., 2015; Ilavenil et al., 2016; Soundharrajan et al., 2019; Diguță et al., 2020; Tatsaporn and Kornkanok, 2020).

### Testing of Probiotic Characteristics

Considering practical applications, some probiotic characteristics of *P. pentosaceus* should also be studied, such as antioxidant and cholesterol-lowering capabilities, or effect in immune response, in addition to the characteristics provided by WHO. A combination of *in vivo* and *in vitro* study can be a strategy.

Evaluation of the antioxidant capacity is taken as an example. *In vitro* use DPPH radical scavenging activity, hydroxyl radical scavenging activity, superoxide anion radical scavenging activity, reducing power assay, assay of antioxidant enzyme activity to determine the antioxidation ability (Zhang et al., 2020a). While *in vivo* study, a murine oxidative damage model was established to study the function and mechanism of oxidative stress resistance in LAB with antioxidant capacity. Some indicators in the tissues (liver, renal, serum, etc.) of the mouse fed LAB (live bacteria or cell extracts), including superoxide dismutase (SOD) activity, glutathione peroxidase (GSH-Px) activity, malondialdehyde (MDA) content, protein amino-compound content, serum endotoxin, structural changes in gut microbial community, were measured and compared with those from the murine oxidative damage model. The significant difference was taken as a reference to evaluate the ability of LAB to intervene oxidative stress *in vivo* (Wang et al., 2020).

As regards the ability to cholesterol-lowering *in vitro*, current study commonly uses the phthalaldehyde method to test the ability *in vitro* and apply the high-fat animal models for evaluation of the ability *in vivo* (Damodharan et al., 2015). Besides, there are some other methods which can rapidly screen the strains with cholesterol-lowering ability in a large strain pool, such as enzyme precipitation method, calcium carbonate plate method (clear circles) method, and indicator method (Vidhyasagar and Jeevaratnam, 2013).

### Requirements in Food Processing

#### Safety

Safety assessment should be performed for the screened *P. pentosaceus* strains. Safety is the primary requisite for probiotics (Singla et al., 2018). *In vitro* tests should cover pathogenicity, toxicity, hemolytic activity, drug sensitivity, antibiotic resistance and so on. The antibiotic resistance of probiotics can be determined by the disk diffusion test (Silva et al., 2017). Hemolysis is the rupture of red blood cells, which may cause sepsis (Thirabunyanon and Hongwittayakorn, 2013). Commonly, blood plate culture is applied to assess hemolysis, where the strains are inoculated onto Columbia blood agar plates using a streaking method and are then cultured at 37°C for 48 h (Diguță et al., 2020).

*In vivo* tests usually adopt animal models using rats, mice or aquatic products, such as fish and shrimp. In addition, the animals used in tests are fed probiotics to generate the experimental group, and the safety of the probiotics will be evaluated by comparing the resistance to different diseases in control group and experimental group, such as immune diseases and conjunctivitis (Arévalo-Villena et al., 2017).

#### Stress Tolerance

As *P. pentosaceus* are prepared for food processing, their stress tolerance and the activity after various industrial processing still needs to be evaluated. Maria de Lourdes Bastos (Rychen et al., 2018) used glucose as a carrier and took diammonium phosphate (5%) and dipotassium phosphate (2.5%) as buffers to standardize *P. pentosaceus* DSM 32291 to 1×1011 CFU/g. The samples were then preserved in aluminum foil bags at room temperature for 18 months without a significant loss of microbial viability.

## Application of *P. pentosaceus*

### Application in Fermentation of Dairy Products

The most common fermented dairy products include cheese, yogurt, cream, etc., which are made from milk (or other milk containing the same milk solids) and fermented by LAB or yeasts. They are nutrient-rich, easy-digestible, palatable, and convenient to storage and are well-received by consumers. *P. pentosaceus* is inherent in or can be processed into cheese, which actively improves the flavor and accelerates cheese ripening, suggestive of a potential cheese fermenter. (Gerasi et al., 2010) isolated 16 strains of *P. pentosaceus* from traditional Swiss Manura cheese and found that *P. pentosaceus* was the only *Pediococcus* in cheese, which is the same as the study by Ilaria et al. (2015). Gerasi identified that *P. pentosaceus* was the dominant flora that contributed to flavor formation and accelerated fermentation in Manura cheese, although it was not dominant in number. Ilaria et al. (2015) isolated 29 strains of *P. pentosaceus* from traditional Italian Marga cheese (TMM) and identified several strains suitable for use in cheese fermentations due to their characteristics, including high-salt tolerance and cryophilic, BSH activity, no biogenic amine (BA) production, rapid acid and conjugated linoleic acid production, and the most important potential in yielding GABA. These characteristics improve the quality and taste of the cheese while being safe and stable, expanding the role of the cheese in promoting consumer health. In the production of yoghourt, *P. pentosaceus* combined with traditional yoghourt fermentation can get better acid production characteristics and contribute to yoghourt products with higher qualities. The study by Balakrishnan and Agrawal (2014) demonstrated that *P. pentosaceus* could make an effective use of the nutrients from goat and camel milk, enhance the antioxidant activity and fatty acid content of the fermented milk, and thereby improve the nutritional value of the products.

The safety issue of dairy products is a concern of the customers. The aflatoxin (AFS) is a harmful component in dairy products, and it may pose a serious potential risk to the health of consumers, especially children the most sensitive to the adverse effects of AFS, due to the heavy consumption worldwide. Martínez et al. (2019) screened out microorganisms that could suppress AFS M1AFM1 into the food chain by adsorption/degradation strategies, and the probiotic *P. pentosaceus* could facilitate the production of metabolites with less toxicity by adsorbing and degrading the AFM1 in milk.

### Application in Fermentation of Fruits and Vegetables

*P. pentosaceus* can be used in fermentation of multiple vegetables to improve the sensory characteristics, make the products safer and more stable in quality, such as radishes, cucumbers, and corns (Kati et al., 2020). The most widespread application is seen in kimchi making. Compared with fresh vegetables, kimchi can satisfy a wider range of tastes and is good at appetite-stimulate appetite. Additionally, it can improve the structure of gut microbial communities due to the abundant functional flora (mainly LAB) and is well-received in the diet of people all over the world, especially in Asia. The kimchi produced by LAB fermentation can overcome the defects of the kimchi naturally fermented, including long fermentation period, susceptible to seasonal changes, unstable quality and short shelf life. Jang et al. (2015) found that *P. pentosaceus* T1 could prevent the overfermentation of kimchi by inhibiting *Leuconostoc mesenteroides* and *Lactobacillus Sakai*. In addition, the kimchi fermented by *P. pentosaceus* T1 could be better accepted in an overall perspective as compared to the kimchi without *P. pentosaceus* T1, accompanied with higher scores in sour taste, kimchi texture, odor and taste, owing to the control of acidity and the number of bacterial cells by *P. pentosaceus*. Moreover, Shukla and Goyal (2014) isolated *P. pentosaceus* CRAG3 from fermented cucumbers and believed that its dextranase and capability of glucan production might have a potential role in functional food applications.

In addition to its host health benefits, *P. pentosaceus* can also improve the quality of fermented fruit and vegetable juice. Xu et al. (2021) used the *P. pentosaceus* derived from fermented cherry juice and pickled porcine ears to ferment cauliflower juice. They found that fermentation by *P. pentosaceus* could change the key odorants and the non-volatile metabolites in cauliflower juice, suggestive of the presence of several metabolic pathways manifested by *P. pentosaceus* and conducive to enhancing the sensory characteristics of cauliflower juice. They also indicated that *P. pentosaceus* could help enhance the sensory characteristics of the cauliflower juice products, contributing to more distinct odor and better tastes.

### Application in Fermentation of Meat Products

Fermented meat products are produced by the bio-fermentation technology, by which raw meat is exposed to specific microorganisms to induce acid or alcohol-production (reduce pH), and then dehydrated by low-temperature separation method. The fermented meat products always have a low pH value, which can inhibit the growth of spoilage/pathogenic bacteria and the production of toxins, thereby contributing to maintaining the drying of the meat products and prolonging the preservation period. In addition, the microbial fermentation of the meat can remove or reduce the unpleasant smell of the meat, which is readily acceptable by consumers. Nanasombat et al. (2017) used agar diffusion test to screen the LAB with antibacterial activity from raw meat and fermented pork products, in an attempt to find the suitable LAB for meat fermentation. They found that *P. pentosaceus* P0805 was the best of the 174 isolates. Further experiments revealed that P0805 has some desirable characteristics, such as the ability to produce inhibitory substances against *Salmonella typhimurium*, catalase to remove the accumulated hydrogen peroxide in fermented products, and nitrate reductase which may facilitate the fermented meat in pinkish. Besides, it does not produce amino acid decarboxylases, and may not result in the accumulation of biogenic amines. These characteristics allow the type of bacterium to be a qualified candidate for good meat fermentation. Sun et al. (2019) purified and analyzed the biochemical characteristics of the *P. pentosaceus* protease isolated from Harbin dry sausages, and proved that *P. pentosaceus* can be used as a starter or enzyme-producing strain of Harbin dry sausages. *P. pentosaceus* can also be combined with other lactic acid bacteria to make a mixed starter to play a better role. Zhang et al. (2020b) prepared the *Lactobacillus curvatus* LAB26 and *P. pentosaceus* SWU73571, which were isolated from traditional sour meat, into fermenters for sour meat processing. The prepared fermenters increased the total number of bacteria, LAB, amino nitrogen and free amino acids, and improved the color of the sour meat. Besides, the water activity and pH value were reduced, accompanied by decreased numbers of coliforms, nitrite, biogenic amine, volatile basic nitrogen and malonaldehyde. Compared with natural fermentation, such fermentation significantly improved the quality and safety of fermented sour meat. Ratanaburee et al. (2013) found that fermenters could significantly increase the GABA content in Nham, contributing to a unique Nham product which was low in fat, carbohydrate and energy, and had the best sensory evaluation.

### Application in Fermentation of Pasta

The yeast fermentation made by *P. pentosaceus* is potentially effective in developing fermented pasta. Montemurro et al. (2020) identified *P. pentosaceus* OA1 and S3N3 from a variety of fermenters according to the acidification growth property and strong proteolytic activity (TFFA increased by up to 80%). Using the strains for bread fermentation, they found that the phytate degradation rate was as high as 58%, and the phenol content and clearance activity were, respectively, increased by 4 times and 2 times, which confirmed the potential of *P. pentosaceus* as a yeast fermenter for bread fermentation. (Plessas et al., 2020) isolated *P. pentosaceus* SP2 from kefir grains, and proved its potential as a yeast fermenter for bread fermentation. They noted that the breads fermented by *P. pentosaceus* SP2 were superior to the yeast breads (wild flora) on market produced under the same conditions as regards acidity, organic acid content and anti-spoilage. Gong and Qi (2020) screened *P. pentosaceus* from Chinese Laomian, and deeply understood the fermentation effect of *P. pentosaceus* in Chinese Laomian, which is conducive to the development of traditional Chinese Laomian. Research by Jin et al. (2020) carried out the separation of anti-fungal lactic acid bacteria and yeast strains, and the isolated strains will be used for starter culture to develop preservative-free yeast bread with improved quality. It was discovered that the combination of *P. pentosaceus* and *Saccharomyces cerevisiae* is a promising yeast starter for making high-quality preservative-free bread. Xing et al. (2019) evaluated the application potential of lactic acid bacteria from traditional sweet and sour dough in different regions of China as raw materials in the production of steamed bread and found that *P. pentosaceus* has applications in improving the quality and quality of steamed bread.

### Some Other Potential Applications

Despite the above mentioned, *P. pentosaceus* has some other applications in the food industry. For example, Raghavendra et al. (Raghavendra et al., 2011) isolated *P. pentosaceus* CFR R123 with phytate degradation ability from fermented grains, and used it in MFSC and soy milk, showing the potential of *P. pentosaceus* CFR R123 in decreasing phytate level and increasing the bioavailability of some minerals (Calcium, Magnesium) and the potential as a fermenter for development of multiple functional fermented grains. Laitila et al. (2006) found that the fermenter containing *P. pentosaceus* promoted the growth of yeasts and inhibited the growth of harmful bacteria during malt fermentation. They also noted a positive effect on malt characteristics, such as the reduction of wort viscosity and β-glucan content, and the enhancement of xylanase and microbial β-glucanase activities, suggestive of improved wort-filtering wheat performance. Toe et al. (2019) isolated 8 strains of LAB from Malaysian food and tested their extracellular proteolytic activity and amino acid production capacity. Since amino acids are important in the growth, reproduction and maintenance of organisms, it is significant to find safer food-grade AA producer strains. The extracellular proteolytic activity of LAB has a great potential for producing functional amino acids due to its involvement in the hydrolysis of extracellular protein molecules into free amino acids. Cui Jin Toe et al. found that the *P. pentosaceus* UL-6 isolated from the 8 strains was highly capable of proline production, and the *P. pentosaceus* UP-2 could produce a series of amino acids with the highest concentration.

## Probiotic Characteristic of *P. Pentosaceous*

### Suppressive to Food-Borne Pathogenic Bacteria

The food-borne diseases caused by food-borne pathogenic bacteria are a global public health problem with growing concerns. Fresh meat and meat products, milk and dairy products, aquatic products and vegetables, etc. are the main bodies with bacterial contamination. *P. pentosaceus*, which has a good ability to against food-borne pathogenic bacteria, has shown a bright future for applications in food industry. It can be used as a biological preservative in processed food and can be an alternative to replace or reduce the use of chemical preservatives. Ghosh et al. (2019) isolated *P. pentosaceus* GS4 (MTCC 12683) from a type of Indian fermented food Khadi. They applied bilayer diffusion method to test the antibacterial and antagonistic abilities of *P. pentosaceus* GS4 on *Staphylococcus aureus*, *Escherichia coli*, *Pseudomonas aeruginosa* and *Listeria monocytogenes*, which identified good suppressive effects on the growth of the tested bacteria. Ladha and Jeevaratnam (2020) isolated *P. pentosaceus* from rumen fluid of goat and investigated the property of the bacteriocin LJR1. LJR1 is a class IIa bacteriocin, which is heat stable (121°C, 30 min), and has strong killing effect on multiple antibiotic-resistant food-borne pathogenic bacteria. Yin et al. (2020) isolated *P. pentosaceus* L1 from pickled radishes and found that it could effectively adhere to small intestinal epithelial cells and suppress the adhesion of ETEC, showing a potential in control of ETEC infection. Cavicchioli et al. (2019) isolated *P. pentosaceus* ST65ACC from raw milk cheese and proved its clearance effect on *Listeria monocytogenes* by using the co-aggregation assay. Cavicchioli et al. (2019) and Muhialdin et al. (2011) assessed the safety and thermostability of *P. pentosaceus* ST65ACC and Te010 and confirmed their use in the control of food-borne pathogenic bacteria as beneficial strains.

The antibacterial mechanisms of *P. pentosaceus* mainly include four aspects: (1) Secrete bacteriocin to damage cell wall or directly kill pathogenic bacteria; (2) The secreted organic acids can infiltrate the cell membrane of pathogenic bacteria and reduce the intracellular pH value to suppress metabolism, while the decrease of pH value can lead to inhibited gene expression of virulence factors; (3) Compete for the adhesion sites on intestinal epithelial cells with pathogenic bacteria to suppress the adhesion of pathogenic bacteria; (4) To aggregate with pathogenic bacteria to make them unable to exert a role.

### Antioxidant Function

Oxidative stress and injury are closely related to accelerated aging and the development of a variety of systemic and metabolic diseases in the body, such as cancer, diabetes, hypertension, and atherosclerosis (Yamamoto et al., 2019). Since the health is growing important and receiving increasing attention, antioxidant studies are prominent in fields like Food Science, Medicine, and Life science. There are some studies reporting that *P. pentosaceus* with antioxidant activity helps improve the antioxidant function of fermented products. Additionally, research believed the application of fermenter YK provides a new functional food, which can decrease the risk of cardiovascular disease caused by oxidative stress (Kim et al., 2019). (Yang et al., 2020) evaluated the antioxidant capacity of Black Gamju extract fermented by *P. pentosaceus* by ABTS radical scavenging assay and β-carotene bleaching assay, and found that ABTS radical scavenging effect was significant. Huang et al., (2020) found that *P. pentosaceus* B49 alleviated oxidative stress in constipated mice by reducing serum malondialdehyde (MDA) levels. Wang et al. (2020) found *P. pentosaceus* PP04 effectively improved oxidative stress induced by high-fat diet by activating Nrf2/CYP2E1 signaling pathway. The signal pathway can be enhanced superoxide dismutase (SOD) and glutathione peroxidase (GSH-px) and antioxidant activity.

The antioxidant mechanisms of *P. pentosaceus* mainly fall into four aspects: (1) Clearance of radicals, including DPPH, hydroxyl radical, and superoxide anions. (2) Tolerance to oxidative stress, mainly O2 or H2O2 at a certain concentration. (3) Ability to anti-lipid peroxidation, which is mainly reflected by the inhibition rate of linoleic acid peroxidation or the content of malondialdehyde (MDA), a product of lipid peroxidation. (4) Equipped with enzymatic and non-enzymatic antioxidant defense systems which can produce antioxidant substances, such as superoxide dismutase (SOD), catalase (CAT), glutathione reductase (GR), glutathione peroxidase (GPx), and glutathione S-transferase (GST).

### Cholesterol-Lowering Function

Cholesterol is an important component of the body tissue characterized by a variety of functions, yet an increase of blood cholesterol is the main risk factor of coronary heart disease (Kumar et al., 2012). Studies have shown that *P. pentosaceus* can be used in development of cholesterol-lowering functional foods after appropriate clinical trials in human beings. Damodharan et al. (2015) established an atherosclerosis diet-induced hyperlipidemia model in male mice (C57BL/6 J) and provided 3 × 108 CFU *P. pentosaceus* KID7 by oral gavage after 28 days, once a day for 32 consecutive days. They found that there was a significant decrease of total cholesterol, low-density fatty acids, and alanine aminotransferase in serum, and total cholesterol in liver, along with a significant increase of free cholic acid content in feces. Wang et al. (2020) fed C57BL/6 N mice with high-fat diet and treated with *P. pentosaceus* PP04. The results showed that the weight gain of total cholesterol (TC), triglyceride (TG) and low-density lipoprotein cholesterol (LDL-C) in mice was significantly reduced, and similar phenomenon was also found (Zhao et al., 2012).

The mechanisms about the cholesterol-lowering function of *P. pentosaceus* may include: (1) The co-precipitation of free cholate and cholesterol; (2) Deconjugation effect on cholate; (3) Cholesterol assimilation to reduce cholesterol content; (4) Simultaneous co-precipitation and absorption.

### Immunomodulatory Function

Owing to the wide applications in the food industry, *P. pentosaceus* plays a certain role in improving people’s health and life quality *via* exerting regulatory functions in immunity. It is reported that *P. pentosaceus* is important in the improvement of immune function and anti-infection ability of the body. Shin et al. (2016) isolated *P. pentosaceus* KFT18 (PE-EPS) from Korean kimchi and found that its extracellular polysaccharide could stimulate IFN-γ to activate macrophages and primary splenocytes, leading to activation of immune response and improvement of the immunosuppression induced by cyclophosphamide. Jonganurakkun et al. (2008) found that there was a significant increase of IFN-γ and IL-12p70 secretion level and a suppression on IL-4 production by the presence of *P. pentosaceus* in spleen cells of mice sensitized with ovalbumin (OVA). It suggested that *P. pentosaceus* could effectively stimulate immune activity and be suppressive to anergy.

The mechanisms of *P. pentosaceus* in immune regulation could be described by (1) the enhanced phagocytosis and natural killer cell activity; and (2) regulating the expression and secretion of cytokines produced by immune organs or cells.

### Cancer Treatment

Cancer is the disease with the highest mortality in the world. Studies have shown that *P. pentosaceus* plays an anti-tumor effect by significantly inhibiting the proliferation of cancer cells. Byung Chull An et al. (2019) developed a gene expression box that can induce a large amount of P8 protein secreted by *P. pentosaceus* SL4 (PP). Through studies, it was found that P8 protein can be used as a therapeutic anti-cancer molecule for colorectal treatment. It can inhibit cell proliferation to a certain extent and reduce the total amount of Cyclin B1/ CDK1 p21 in a P8 dose-dependent manner. Furthermore, Shukla and Goyal (2013) showed that the dextran isolated from *P. pentosaceus* CRAG3 has anti-cancer properties, and has reduced activity on both HeLa and HT29 cell lines, which may be related to the adhesion disorder caused by the ability of dextran to modify tumor cell membrane surface proteins. Thirabunyanon and Hongwittayakorn (2013) isolated a strain of *P. pentosaceus* FP3 and found its inhibitory effect on the proliferation of colon cancer cells. Meanwhile, it was speculated that *P. pentosaceus* FP3 adheres to colon cancer cells and induces the biological production of short-chain fatty acids (SCFAs), thereby inducing the apoptosis of colon cancer cells is an important mechanism.

The specific mechanism remains an open issue. Speculations include: (1) Induce tumor cell apoptosis by secreting organic acids; (2) Inhibit cell spreading and metastasis *via* producing glucan, which can modify membrane surface proteins leading to decreased adhesion of tumor cells. (3) Induce *P. pentosaceus* to synthesize P8 protein and acting on cyclin to inhibit cell proliferation.

## Summary and Conclusion

As for *P. pentosaceus*, there are still many problems which need to be solved. For example, the strains documented in NCBI are mostly isolated from fermented foods or animals and plants (Jiang et al., 2020). There is a notion that probiotic bacterial strains should be derived from the human gut microbiome, which may help easier intestinal colonization and improve more specific applications. According to the complex interactions between the gut microbiome and their multi-faceted effect on the host, the isolation of *P. pentosaceus* from the human gastrointestinal tract is also worthy of being studied in the future. Besides, studies devoted to the interactions of *P. pentosaceus* with the host intestinal microbiome are a few, leading to the functions related to gut microecological balance regulation less evidenced (Suez et al., 2019).

Another problem is that, there is a deficiency in *in vivo* experiments for the probiotic *P. pentosaceus*. Most of the studies only covered the screening of available bacterial strains and identification of the probiotic characteristics, without genomics research or further *in vivo* study. Besides, the mechanisms about the probiotic effect of *P. pentosaceus* have not been thoroughly studied. For instance, some strains in murine diarrhea models were found to aggravate the inflammatory response by promoting the production of pro-inflammatory factors, suggestive of high strain specificity which requires further *in vivo* verification (B. et al., 2017). There are some studies reporting that the *P. pentosaceus* derived from food may also potentially risky. Møller et al. (2020) found that the *P. pentosaceus* isolated from cheese were capable of producing histamine. This was the first report on *P. pentosaceus* as a histamine-producing bacterium. High concentrations of histamine may cause symptoms, such as urticaria, rash, hypertension, and headache.

To sum up, the current studies on *P. pentosaceus* are not perfect. *P. pentosaceus* as a normal flora in gut microbiota plays a role in antioxidation, cholesterol-lowering, immune regulation, and cancer treatment. Besides, *P. pentosaceus* can be a promising natural additive in food processing, which can help improve product quality and safety while assisting fermentation. It also indicates that *P. pentosaceus*, as a potential probiotic bacterium prevalent in traditional food, is promising for future applications. In the future, a thorough understanding of the functions and related mechanisms of *P. pentosaceus* at the molecular level is required, in an attempt to make it further promoted in a variety of fields and play a more active role.

## Author Contributions

JY and YQ: conceptualization. LH: methodology. YZ: resources. YQ: writing – original draft preparation. LH, YZ, JiX, and JuX: writing – review and editing. WL and DZ: supervision. JY, QT, and DD: project administration and funding acquisition. All authors have read and agreed to the published version of the manuscript.

## Funding

This research was funded by the National Natural Science Foundation of China (31700004), Shandong Key Research and Development Program (2019JZZY010724), Construction of Innovative Provinces in Hunan Province (2019RS3022), and the National Students Platform for Innovation and Entrepreneurship Training Program (2020056). The funding bodies had no contribution in the study design, data collection, interpretation, or preparation of the manuscript.

## Conflict of Interest

JiX and DD are employed by Tangrenshen Group Co., Ltd., China.

The remaining authors declare that the research was conducted in the absence of any commercial or financial relationships that could be construed as a potential conflict of interest.

## Publisher’s Note

All claims expressed in this article are solely those of the authors and do not necessarily represent those of their affiliated organizations, or those of the publisher, the editors and the reviewers. Any product that may be evaluated in this article, or claim that may be made by its manufacturer, is not guaranteed or endorsed by the publisher.
